# Life conditions and dental behavior as risk factors for noma among children in Niger; preliminary results

**DOI:** 10.1186/2047-2994-4-S1-P259

**Published:** 2015-06-16

**Authors:** D Baratti-Mayer, A Gayet-Ageron, D Pittet, and the GESNOMA

**Affiliations:** 1 University of Geneva Hospitals, Switzerland

## Introduction

Noma is a devastating facial gangrene affecting young children in the developing world. We previously showed that the disease was associated with an imbalance in the oral microbiota, with chronic malnutrition, recent febrile illness, large households and short intervals between pregnancies in the mother. [1] Necrotizing gingivitis was postulated to precede the occurrence of noma.

## Objectives

To describe oral health, sociodemographic and nutritional habits among children living in a village with a past history of noma (“noma-village”, NV) or in a village with no past noma cases (“non-noma village”, NNV).

## Methods

Cross-sectional study during a one-month period in March 2014 among children aged <12 years randomly selected=“selected” from 4 (2NV; 2NNV) villages in the Zinder region (Niger, Africa). The four villages were not different regarding size, culture, and were equally distant from medical facilities. All children received a dental and physical examination and their legal representatives answered a complete questionnaire about sociodemographic, environmental and behaviours’ data.

## Results

A total of 440 children (110/village) of a mean age 43 months (±SD 14) were included (52% were girls). Half of the children had moderate to severe stunting (n=200) and a third had moderate to severe wasting (n=121). Median number of persons in the household was 7 and two thirds of the children were > 4^th^ in the sibling order (n=260). Median number of past pregnancies in the mothers was 5 and median time from the last pregnancy was 2 years. Children living in the 2 NV had a significantly lower height-for-age z-score defining stunting (OR 0.84: 0.72 to 0.98,) compared to those living in NNV, a smaller arm circumference (OR 0.77: 0.62 to 0.95), were more frequently >5^th^ in the sibling order (OR 2.30: 1.39 to 3.82), were less often exclusively breastfed for a 4-6-month duration (OR 0.30: 0.17 to 0.53) and used more frequently a mix of water, sand or water and coal for their dental care than water alone (OR 11.38: 2.81 to 46.07).

## Conclusion

Life conditions and dental behaviors of children living in NV were different from those living in NNV, reinforcing the theory of environmental triggers explaining a differential incidence of noma by geographical areas in the region of Zinder (Niger).

## Disclosure of interest

None declared.
